# Bibliographical Notices

**Published:** 1839-04-01

**Authors:** 


### Periscope;

OR,

### CIRCUMSPECTIVE REVIEW.

" Ore trahit quodcunque potest, atque addit acervo."

### Notices of some New Works.

THOUGHTS AND OBSERVATIONS UPON PAUPERISM, POOR LAWS, EMIGRATION, MEDICAL RELIEF, AND THE PREVENTION OF CRIME. By *William Fergusson*, M.D. F.R.S.E., Inspector General of Army Hospitals. 8vo. London, 1839.

THE substance of these Thoughts first appeared in the shape of letters in the Windsor Express. The object of the author is evidently to improve the social condition of the lower classes in this country, and to lend his hand to extricate them from that degraded sink of pauperism into which they had fallen. Dr. Fergusson's opinion of the old poor law and its consequences may be gathered from the following passage :—

" The compulsory rate answered temporarily the *same* purpose in England, but its operation here, as everywhere else, infallibly led to the same ruin ; and our beautiful land was but very lately in the speedy course for becoming a vast pauper warren, where the demoralized wretch, in order to share in the plunder, was obliged, in the first instance, to divest himself of every vestige of property—to convert his comely cottage into a hovel—and to assume the habits and habiliments of beggary ; for the practice of any of the decencies of life would immediately have subjected him to robbery, and constituted him a giver instead of a partaker in the spoil. The rural population was everywhere plunging into this stye ; beggary insatiate and insatiable had become almost the sole vocation of the man, and the trade of bastardy, so lucrative and so strenuously advocated by good, but mistaken men—the resource of the woman. Some of the most fertile districts of our country had actually lapsed into this shocking state ; rent had ceased to be paid, or the land in consequence to be cultivated with effect ; they would have multiplied like the beasts of the field, and like these beasts they would have come in the process of subdivision, and in the scramble for a share, as we see in the present peasantry of Ireland, to have destroyed one another. And this is the state of things preached up and advocated by the charity-mongers of the present day,—while there exists a most corrupt and venal press which, with persevering malignity, strives to stir up the people in insurrection against this charter of their worldly rescue—to make it a question of political party—to array against it not only all the feelings of the uneducated poor, but to use it as an instrument of party hostility against their rivals, in the struggle for the attainment of political power."

It is impossible for any unprejudiced man, possessed of sound reasoning powers, to peruse the report of the poor law committee, and not to perceive that the old poor law was working the degradation of the lower classes of this country, and the injury, if not the destruction, of all. A bonus was offered on the spread of pauperism and of all the vices that follow in its train. When the man who did not work was as well off as the man who did, and the female who had lost her virtue and could count her bastards was in a better situation than the virtuous maid, or the wife or widow with her family, it might easily be predicted that laziness would prevail and that prostitution would flourish. Yet this is the state of things which some would affect to consider the beau ideal of moral and political wisdom, and the attempt to remedy it is denounced as the extreme of tyrannous cruelty. As Dr. Fergusson observes—

"Habits of virtue go hand-in-hand with industry, and of these economy and providence are, in a worldly sense, the first. A code of laws, therefore, striking at the very root of the last, made their practice all but impossible. We established, indeed Savings' Banks to receive the deposits of the careful; but the law in substance said—Why should you be so foolish as to make them?—take your gains to the pot-house—waste them in any sensuality you please—the parish is your resource and inheritance—is bound to take your children off your hands, to relieve you from your obligations as parents, and to parents—to guard you in sickness, and support you in old age—you have only to shew, that whatever may have been your occasional gains, you have ever been improvident and reckless, and have resolutely abstained from laying by a single farthing; resort then to her, for she acknowledges no disqualification but a decent garb and habits of virtue! Here let me pause to express my admiration of the intrinsic worth of the English national character, which not even such a system of law could irremediably vitiate or destroy."

We all know the outcry against the Union Bastilles. But this may be said with safety—that if we give the man who will not work the same comforts and the same freedom as are earned by the sweat of the brow, the majority will be found to decline labour and to live on alms. Out-door relief to the able-bodied must generate idleness and all the evils in its train. Men must look upon pauperism as an evil, or they will not struggle to avoid it.

But it is by no means equally clear that out-door relief might not be extended with advantage to the sick and to the aged. The "out-and-out" economists scout, we know, such an idea. According to them the labourer ought to have laid by sufficient money during the vigour of life, to support him in sickness, or when too old for work. But when we reflect on the low rate of wages, particularly to agricultural labourers, and the occasional deficiency of work even for those who are most willing to engage in it, we must pause ere we subscribe to such a doctrine. It seems to us impossible that the savings of such persons can ever amount to sufficient to keep them in sickness and senectitude.

Much of the antipathy to the New Poor Law has arisen from the assumption of offensively arbitrary power by the Commissioners. The change from the old system to the new was perhaps too sudden and violent. A law which necessarily wears the appearance of harshness, was made in practice harsher. The feelings of Englishmen revolted against the semblance of dictation and oppression, and the final success of the experiment has been endangered.

Dr. Fergusson warmly recommends emigration, in connection with the improved system of poor laws at home. The friends of the poor are in excessive dudgeon if they (the poor, not their friends) are not permitted to starve or to pauperise in England, and in England only. What is universally done in a small way, they will not permit to be done in a large. If a street has too many bakers the obvious remedy is for some to remove, and to find a neighbourhood in which dough is in demand. Every body acts upon this hint. But if a country has too many inhabitants, those who are too poor to transport themselves elsewhere, are not to be transported at the cost of the rest. This is exile, white slavery, and we know not what. Nature has limited the size of our island, but she has not limited the birth of human beings on it. According to these friends of humanity we are to go on procreating and pauperising to the end of the chapter, and to make no attempt to find other provender for the superfluous mouths.

We pass over much of Dr. Fergusson's pamphlet, occupied as it is with general political questions, and proceed to what particularly affects ourselves—the present system of administering medical relief to the poor. A villainous system enough. We cannot help thinking that the hard measure doled out to medical men, has partly been due to their mixing themselves up with the general opposition to the New Poor Law. It is well known that some of the medical journals have displayed a very strong hostility to this measure. The majority of the Legislature, and indeed of all intelligent men in the empire, are so strongly convinced of the necessity for such a law, and so opposed to the clamour against it, that any body of men mixed up in that clamour are certain to be viewed with distrust and aversion. The claims of the medical profession were not likely to be heard with favour, if mingled with a medical anti-poor-law shout.

Dr. Fergusson observes:—

"Centralization—the *vis unita fortior*—I acknowledge to be an excellent principle in all government, but it is not applicable to medical attendance upon the poor; when the Poor Law Commissioners therefore united a number of parishes together and then advertised for the lowest tenders, calling up the inexperienced tyro from the schools, in want of a place, to underbid the established practitioner who had long been in charge, they handed over the sick poor to serve the purpose of the former's selfish speculation, or held him up *in terrorem* over the latter, with the view of imposing conditions upon him which he never could execute in fairness either to the unfortunate paupers or himself."

Certainly the system of "tender," is any thing but a tender system for the poor. Would these fat and insolent Commissioners provide medical relief for their own bloated carcasses on tender? Would they advertise for the lowest doctor—the cheapest physic? Catch a weasel asleep! But they thought, no doubt, that the worst would do for the poor, and that the parish doctor was a sneaking animal, and would be bullied with impunity. The Commissioners have already found that the medical profession will not tamely submit to be injured and insulted in the same breath.

The great remedy is union, association, and a common principle and mode of action on the part of the profession itself. If it *is* united, neither commissioners nor ministers, neither the public nor the executive, can trample on it. If broken, jealous, disunited, all will prey on it. We recommend then the formation of associations, their extension, their co-operation. Such associations will present a bold front to assaults from without, and (what is of consequence) will repress treason within. The profession has suffered because its own members have been tempted to betray its interests, in the eager search of their own.

Dr. Fergusson proceeds:—

"The system of tender, in any shape, for personal services is a vile one. If acted upon extensively it would destroy any profession, and has been most unwarrantably applied in this case to the general practitioners of England, comprehending a body so numerous that they may be reckoned by thousands; and ever since the passing of the Apothecaries' Act, in the year 1815, have been the best qualified and most valuable that any country ever possessed; search Europe through, there is nothing to be found at all comparable to them. There is not a family in the land that has not been beholden to them for the lives of its members, and to make war upon such men for the sake of a show of economy —for, as I shall presently prove, it can be nothing more, if it be even that— may almost be characterised as national ingratitude. Whoever declaims in this manner ought to be prepared to point out a remedy, and I shall here propose a very simple, and I believe, a perfectly practicable one. Let every pauper on the union list, sick and well, be rated at five shillings per annum, for medical relief; that is, for a small fraction more than a penny a-week, and let this be the pay of the best qualified, and if possible, the nearest practitioner; for the nearest, for obvious reasons, must always be the best. There can be little doubt that the will accept the contract, for the penalty of having his practice invaded by another would immediately follow. No one residing at a distance should be considered eligible to the trust, for if the poor lie beyond his beat, they will infallibly be neglected, because he will be more lucratively employed nearer home, and no recompense that the union funds could afford to give, would remunerate him for the sacrifice. It is, then, most essential that the sick poor should be within the compass of his daily rounds, for then his knowledge of the pauper's character, and responsibility for his own, will insure his attendance and detect imposture. The proximity (surveillance) of his better patients will constitute them guardians of the poorer, and the expense and trouble to their medical attendant will comparatively be as nothing. This, surely, is what ought to be, but what is it in reality?"

We recommend this pamphlet to the consideration of our provincial brethren.

---

Stammering Practically Considered, with the Treatment in detail. By *T. Bartlett*, Assistant-Surgeon in the King's Own Light Infantry. Duodecimo, pp. 84. Sherwood, Gilbert and Piper.

So many methods have, of late, been promulgated for the cure of the unfortunate habit of stammering, each method by its author stated to be infallible, his statement being backed by a multitude of well-authenticated cases, that it appears incredible that a stammerer should ever be met with. But when we reflect on the annoyance caused to the afflicted individual by this troublesome complaint, and the simplicity of the rules generally laid down for its removal, we cannot wonder that the majority of stammerers on having some plan of speaking pointed out to them, from which they find material benefit, should be willing to believe that they have discovered a remedy for their complaint which will be always efficacious, and that they are permanently cured. And when we consider that stammering is an unnatural action of organs so complicated and so little under the control of the will as those of speech, an action too, become habitual by the practice of years, we shall not be surprised, that, in most cases, no long time elapses before the stammerer, to his annoyance, finds that his old habit is beginning to return, and that at times, do what he will, he cannot speak fluently and without effort to himself. He then begins to doubt the possibility of his cure, he ceases to pay attention to his speaking, and speedily relapses into as bad a state as he was in before. When a healthy individual gives utterance to his thoughts, he has no occasion to think about his organs of speech; the instant the mind has determined on the word to be employed, those organs take on the proper action for producing that word. But with the stammerer, when in those situations in which he has been accustomed to stammer, the case is different; his organs of speech instead of taking on the proper action for forming the words conceived in his mind, will, if not prevented by an effort or volition of the speaker, take on the unnatural action become habitual to them. This volition in the heat of conversation or in a moment of surprise he will often neglect to exert, and from excitement or debility of the nervous system, he will sometimes be unable to exercise. It can only be by perseveringly following for no inconsiderable period, and undismayed by partial relapses, the rules laid down, that the stammerer can entirely overcome this disposition in the organs of speech to take on the unnatural action. And, any one who expects to be permanently cured in a short space of time, will find himself egregiously disappointed.

Mr. Bartlett, in the little treatise before us, after some remarks on the inconveniences of stammering, about which, we suppose, there will be little difference of opinion, and a description of the anatomy of the organs of speech, gives the following account of the method in which the various letters of the alphabet are formed.

"*A** is formed by a strong and grave expression of the breath through the mouth, which is open, whilst the tongue contracts itself to the root. *E* is formed by elevating the tongue nearly to the palate and lips: the tongue is, in the formation of this letter, as close to the palate as possible, without touching it. To form *O*, the lips are protruded in a circular form, the corners of the mouth being contracted, so as to produce the *os rotundum*, a picture of the letter it sounds. In forming *I*, the mouth is opened as if to pronounce *A*; and on the first effort of the voice for that purpose, its progress is checked by a sudden motion of the under jaw towards the upper, and by then instantly cutting off all sound. *U* is formed by protruding the lips a little more than for *O*, forming a smaller aperture with them, and, instead of swelling the voice in the middle of the mouth, bringing it as forward as possible to the lips. *Y* final, either in a word or syllable, is a pure vowel, and has the sound of either *e* or *i*. *W* final is equivalent to *oo*.

The consonants are divided into the continuous, sometimes called semi-vowels, or liquids; the explosive, which are produced by a very sudden, quick expiration; and the sibilant, which are formed as if by a mere aspiration. For the two latter, the breath or voice is stopped in its passage through the mouth; for the former it is allowed a free passage, although the apertures are more narrowed than for the vowels. The semi-vowels are *M, N, R, L*. *M* labio-nasal is formed by a compression of the lips, and letting the voice issue by the nose. *N* is formed by pressing the tip of the tongue to the gums of the upper teeth, and breathing through the nose with the mouth open. For *R* and *L*, the front of the tongue is elevated so as to touch the palate just above the teeth; for the *r*, the point is drawn back, so as to allow the air to escape; and for the *l*, the point is firmly pressed against the palate, and the breath escapes by the sides: this is the most vocal of all the consonants. Ben Johnson says that *l* melteth in the sounding, and is therefore called a liquid. This however cannot be the reason that *r* is called a liquid, for no two letters can, in this respect, be more opposite. The explosive consonants are *K, T, P, G, D, B*. *K* is formed by pressing the middle of the tongue to the roof of the mouth, and separating them rather quickly. *T* is formed by applying—when the breath is stopped—the fore part of the tongue forcibly to that part of the palate which is contiguous to the fore teeth. *P* is formed by the sudden opening of the lips, in order to emit the compressed sound of the vowel. *G* is formed like *K*, the tongue and roof of the mouth being separated more gently. By using the guttural sound, when the fore part of the tongue is forcibly applied to the front of the palate, *D* is produced. The guttural sound is that kind of murmur, as Bishop Wilkins expresses it, which is heard in the throat before the breath is emitted with the vocal sound. *B* is formed like *T*, the guttural sound being added. The sibilant consonants are *H, Z, S, V, F*. *H* is the note of aspiration, and is formed in various positions, according to the vowel with which it is combined. If the tip of the tongue be turned up towards the upper gum, so as not to touch it, a space is left between the tongue and the palate for the breath to issue, which forms the sibilating sound of *S*: if this operation be accompanied with a guttural sound, the letter *Z* will be pronounced. *F* and *V* are formed by pressing the upper teeth upon the under lip, and sounding the vowel *e* before the former and after the latter of these letters. *C* is formed either like *k* or like *s*. The English *J* is a double consonant, compounded of *d* and the French *j*. *Q* has always the sound of *k*; it its constantly followed by *u*, pronounced like *w*. *KS* give the

---

* "Vide Richard Payne Knight, Analytical Essay on the Greek Alphabet. 1791.

See also the works of Lowth, Elphinston, Kenrick, Sheridan, Johnson, Walker, Nares, Crombie, and Holder."

sharp and *gz* the flat sound of *X*. When there is a difficulty in pronouncing the lisping consonant *th*, let the person protrude his tongue a little way beyond the teeth, and press it between them as if going to bite the tip of it: while this is doing, if he wishes to pronounce *thin*, let him hiss as if to sound the letter *s*; and after the hiss, let him draw back his tongue within his teeth, and pronounce the preposition *in*, and thus will the word *thin* be perfectly pronounced. It will be proper to make him dwell some time with the tongue beyond the teeth, in order to form a habit, and to pronounce daily many words beginning and ending with these letters."

Mr. Barrett very justly deprecates the employment of any harshness towards a child labouring under this complaint; a little reflexion must convince us, that rendering the child timid and nervous on the subject of his speech, is the very way to aggravate the disorder. He also advises that particular attention should be paid to the state of the stomach and bowels. No one who has seen what a remarkable exacerbation of the complaint an attack of indigestion will occasion, can question the propriety of this advice.

Mr. Bartlett then lays down a series of rules to be followed by stammerers. He says in Rule 1—"Before your commence speaking draw a long breath."

This must not be carried too far; few stammerers habitually begin to speak with the lungs exhausted of air; it is in the attempt to articulate that the air escapes. No doubt, when the stammerer finds that his lungs are getting empty, he ought to stop, and then he will naturally refill his lungs with air.

Rule 2, is—"That great attention should be given that the lips, teeth and tongue, perform their different functions when employing the letters requiring the individual use of them."

Rule 3, is—"Lay a decided stress or accent on the last syllable of every word."

This rule we think a very important one, as no one who follows it can speak quickly, and "speak slowly" may be called the golden rule for stammerers.

Rule 7, is—"Be certain of what you are about to say."

This rule requires no comment.

Mr. Bartlett's 12th and last Rule—"Release the mind of all fear and trepidation"—is a very good rule, but unfortunately, impracticable. Give the patient confidence that he can speak without impediment, and he will speak without fear, but until then no mental effort of his can remove his nervous feeling on the subject.

We have now stated the substance of the remedies proposed by Mr. Bartlett for stammering; though not particularly new, they are in accordance with common sense; and though the book might well be compressed into a third of its present dimensions, we think an adult who labours under this malady, or the parent of a child so afflicted, will not be sorry for purchasing this little work and attentively perusing it.

---

An Improvemrnt in the Pathology and Treatment of Small-Pox, &c. By *Robert Stephens*, M.R.C.S. 8vo. pp. 24. Renshaw, Dec. 1838.

This little essay was read before the Medical and Chirurgical Society some two years ago, but not published in the Society's Transactions, probably for the reason adduced by the author himself—namely, "that the subject was too abstruse to be caught at a glance." This, however, ought to have been a reason for the publication, in order that the public might have time to read, mark, and inwardly digest the subject of the thesis. We apprehend that the following passage contains the pith of the pamphlet, and the principle of ameliorating small-pox.

"As Dr. Mason Good observes, the severity of the precursory symptoms and of the eventual disease, hold a pretty accurate balance. Now the precursory symptoms are febrile and excited action, in order to render the superficial tissues fit for the development of the eruption. Can no reason be given why this febrile and excited action should be greater in one individual than another. Surely temperament and excitability, either temporary or otherwise, should not be put out of the question. This is the sole cause, though it may sometimes appear otherwise to a superficial observer. That there is a specific and *unaccountable* aptitude, is an unsupported opinion, and an idle attempt at rule and definition which is so baneful a principle to the science of medicine. This notion at once closes the path of enquiry, whilst by adopting the opinion that excitability and temperament are the causes of occasional malignancy, it must be remembered that science and ingenuity can control excitability, and consequently the diseases it aggravates. This is a most valuable pathological basis.

It is to be remarked, that the most delicate persons are often the most susceptible, and liable to violent inflammatory excitement.

When small-pox proves fatal, the immediate cause is, that typhoid symptoms and exhaustion of the nervous or functional power have supervened upon undue excitement, and where the fever has run very high, the patient being delicate and excitable, with little nervous or vital power, this has taken place even in consequence of the precursory fever, before the eruption is complete. In this case we usually have typhus with purple spots,* which might always be prevented by controlling the excitement in the first instance by using decided measures. More frequently when small-pox proves fatal, it is at the latter part of the disease, exhaustion supervening upon the excessive inflammation which attends the suppuration of a full eruption, just as in the case of an extensive burn or scald. The vital power is during these states of inflammatory excitement used up, or burnt out too quickly as it were. Its undue expenditure at first constitutes the severity of the precursory symptoms which is always followed by a full eruption; and, withal, the patient is left the less capable of standing against the excessive inflammation which is sure to accompany the ripening of a full eruption. Therefore the grand opportunity is at the commencement of the disease, when by controlling the excitability, not only the nervous power will be kept in reserve, but a light form of small-pox will ensue."

The tartrate of antimony is the favourite remedy of the author, and preferred to venesection.

"By keeping down the cutaneous circulation, the appearance of the eruption is delayed for a time—but all this is completely in the control of the medical attendant, if he have any ingenuity; and by giving calomel, or the blue pill with the tartrate of antimony, I have much reason to think this delay is beneficial, because it promotes the decomposition of the virus which is already in the system."

Some cases are appended to illustrate the utility of keeping the cutaneous circulation in check during the eruptive fever.

---

The Philosophy of Disease, &c. By James Bower Harrison, M.R.C.S. Small 8vo, pp. 152. Simpkin and Marshall, 1838.

We have now three philosophies—one of health, one of life, and one of disease. We only want one more—the philosophy of the last act of the drama. And we need this fourth philosophy much more than either of the other three. When the curtain of life is about to drop, and the last lingering look to be taken

* "From relaxation of the exhalents and tenuity of the circulating fluids."

of the cheerful day and all we hold dear on this wondrous globe—it requires even more than philosophy to enable frail man to contemplate with composure the approaching gloom of eternal night!

The little essay before us appears to have small connexion with philosophy of any kind. It presents a light sketch of almost every disease to which mankind is liable—but especially of inflammation—and is addressed to the general reader. It is manifest that these sketches are too slight to satisfy the medical practitioner or student—and we doubt very much whether they are calculated for non-professional perusal. Light as are the portraits, they will be found too heavy in technicalities for general readers, who are more taken up with Nicholas Nickleby, or Oliver Twist, than with the philosophy of that which they cordially hate, as conjuring up recollections and associations of the most triste and nauseating kind—mal-odorous pills and bitter draughts—the tenacious leech and the burning blister. The hypochondriac, who is the principal purchaser of popular works on medicine, will here be distracted by such a multiplicity of maladies, that he will hardly be able to pick out the one that suits his case—so little detailed are the symptoms, and so evanescent are the features of each particular form of disease. It is true that Mr. Harrison has run a kind of glossary through his pages, exhibiting the Greek and Latin derivations of the principal terms; but to the learned reader these are unnecessary, and to the unlearned they are—"Greek and Latin"—that is to say, they are unintelligible. However, if Dr. Harrison can enlighten the popular mind, and can teach it to appreciate the blessings and the abilities of practitioners, we have not the slightest objection. We wish him every success.

---

Vital Statistics of Glasgow. By *Robert Cowan*, M.D. one of the Physicians of the Glasgow Royal Infirmary. 8vo. stitched, pp. 54, 1838.

This is a valuable pamphlet, and reflects great credit on the zeal as well as the ability of Dr. Cowan. The pamphlet is occupied with:—

I. Statistics of Fever and Small-pox prior to 1837.
II. Statistics of Fever for 1837.
III. Remarks suggested by the Mortality Bills.

It would be "laterem lavare" to analyse a pamphlet of this description. Consisting of numerical tables, and of deductions from them, condensation is impossible. We shall conteut ourselves with selecting some of the more prominent points, and endeavour to put our readers in possession of what is most calculated to interest or to instruct them.

It appears that, in 1791, the population of Glasgow was 66,578—in 1831, 202,426, an enormous increase. That increase has arisen in a very great degree from immigration, and from the increased demand for female domestic servants, and for female labour in the numerous cotton and power-loom factories and bleachfields in the neighbourhood of the city; a large proportion of the immigrants have been females. Those who resort for employment to towns are generally from the age of fifteen to twenty-five, a fact of some importance in reference to fever.

In 1819, there was one Irish person out of every $9\frac{67}{100}$ of the inhabitants; and in 1831, one out of every $5\frac{69}{100}$. From this increase of Irish alone, without including the influx of labourers from the Highlands and Lowlands of Scotland, it is quite obvious that the relative proportion of the middle and wealthier classes to the labouring class must have been yearly diminishing; and, hence, one source of the increasing rate of mortality in Glasgow.

The present accommodation in the Glasgow hospital amounts to 450 beds. Temporary hospitals have been required and provided on several occasions.

The following facts shew in a striking manner the increase and the alarming prevalence of fever in Glasgow. On dividing the period between the years 1795 and 1836 into septenniads, it appears that, in the first septenniad, the fever patients treated in the Infirmary were—

| | | |
|---|---|---|
| | 12.92 | per cent of the whole. |
| In the second, | 9.84 | — — |
| In the third | 8.17 | — — |
| In the fourth, | 31.77 | — — |
| In the fifth, | 36.19 | — — |
| In the sixth, | 49.96 | — — |

and if to this table, strictly applicable to the Royal Infirmary, we add the numbers treated in the temporary hospitals, we will raise the per centage in the fourth period.

From 31.77 to 47.62; and in the sixth period,
From 49,96 to 54.83.

During the first 35 years embraced in the Table the number of patients affected with fever treated in the Infirmary amounts to 11,511, while in the last seven years it amounts to 11,751.

It is on comparing Glasgow with some other great towns that the amount of its fever cases becomes most conspicuous. Take Manchester. This town, with a population at the last census, of 227,808, and which, in its constitution and density must nearly resemble that of Glasgow, has been for years, and is now comparatively free from fever. The average annual number treated in the Manchester Fever Hospital for seven years ending in 1836, was .. .. 497

The annual average in Glasgow during the same period .. .. 1842

The number treated in Manchester Hospital in 1836, .. .. 780

The — — Glasgow — — .. .. 3125

Fever is now diminishing in Manchester, while it is increasing in Glasgow.

The prevalence of fever in Glasgow, when compared with Manchester, is still more strikingly contrasted by the great change which has taken place in this respect. From 1797 to 1806, both inclusive, the number of the fever patients treated in the Glasgow Infirmary was only 883, while those treated in the Manchester Fever Hospital amounted 4618.

" We have proved," says Dr. Cowan, " that since 1816, but more particularly during the last seven years, fever has been steadily increasing in the City of Glasgow, and that its victims constitute within a fraction of 55 out of every 100 patients treated in our hospitals, independently of those treated by the district surgeons within the Burgh.

This increase, especially during the last seven years, has taken place, not in years of famine or distress, but during a period of unexampled prosperity—a period when the wages of labour have been ample—the prices of provisions comparatively low, and every individual, able and willing to work, secure of steady and remunerating employment."

He adds :—

" Many of the causes of the production and propagation of fever must be ascribed to the habits of our population ; to the total want of cleanliness among the lower orders of the community; to the absence of ventilation in the more densely-peopled districts ; and to the accumulation, for weeks or months together, of filth of every description in our public and private dunghills ; to the over-crowded state of the lodging-houses resorted to by the lowest classes ; and to many other circumstances unnecessary to mention."

Dr. Cowan dwells on the almost obvious fact that, amongst the poor, the longer the duration of a malady, the more heavily it presses on them and on the community which must suport them. We cannot therefore but agree with him in the conclusion, that the prevalence of fever in Glasgow presents obstacles to the promotion of social improvement among the lower classes, and is productive of an amount of human misery, credible to those only who have witnessed it.

II. The statistics of the Glasgow Fever Hospital from the 31st of October, 1835, to the 1st of November 1836, next occupy the attention of our author.

The Glasgow Fever Hospital can accommodate, without being over-crowded, two hundred and twenty patients.

From the 31st of October, 1835, till the 1st of November, 1836, there were admitted into the wards of the Fever Hospital, 2655 persons.

In the first six months they were 909, in the last 1746.

The number of patients admitted into the Fever Hospital in 1835, only amounted to 1359, and, on an average of the last eight years, to 1477; while in 1836 the number was 3125, being greater than that treated in 1832, in the Infirmary (1589) and Mile-End Hospitals (1145), 2734.

Some little time ago, a paper was published in the Dublin Journal by Dr. Lombard of Geneva, in which he endeavoured to make it appear that the prevalence of fever in Glasgow is mainly owing to the Irish. We gave an abstract of that paper in this Journal. Dr. Cowan observes in regard to it:—

"The proportion of Irish treated in the Fever Hospital is much less than what is generally believed by those who have not paid attention to the subject. Dr. Lombard of Geneva, estimates the number of Irish resident in Glasgow at 60,000, and ascribes the prevalence, and what he deems the peculiarities of our Fever, to the number of Irish resident in Glasgow. The author of the article "Vital Statistics," in M'Culloch's Statistics of the British Empire, vol. ii. p. 572, makes the following remarks:—'The increasing mortality in Glasgow is no doubt in part due to the accession of Irish population, who amounted, in 1831, to more than 1-6th of the inhabitants. The poor Irish, we strongly suspect, are keeping up, if they be not introducing, the Fevers of their wretched country in the heart of the British cities. This is confirmed in the case of Glasgow, by the ages at which the mortality is augmented, and by a report of the Glasgow Infirmary before us, from which it appears that, in the year 1835, out of 3260 patients treated, 1258 had Fevers, and of these 125 died.'

This statement will be proved to be incorrect while adverting, at the close of this essay, to the influence which Fever has had in augmenting the mortality of Glasgow, especially during 1835, the year alluded to in the quotation."

The population of Glasgow and its suburbs in 1831, was ranked in the Government census at 93,724 males—108,702 females—in all, 202,426.

Dr. Cowan subjoins several Tables. From an examination of them, it appears that the period of life at which Fever is most liable to occur, is from the age of 20 to 25 years for the males, when the proportion is 21.23 per cent. and from the age of 15 to 20 for females, when the proportion is 23.83 per cent.

The number of females at every age prior to 25 exceeds that of the males.

The number of females admitted under 20 years of age is 571

Males .. .. .. .. .. .. .. .. .. 480

Excess of females under 20 .. .. 91 or 8.64 per cent.

This fact must be kept in view, as it has an important bearing on the relative mortality of the sexes.

The number of both sexes admitted from the age of 20 to that of 30 years, is 1216, or 53.87 per cent.

The number admitted under the age of 30 years, is 1766 or 78.24 per cent.

The admissions rapidly diminish after the age of 40. Of 2257 individuals affected with fever, 2075 were under 40 years of age and only 182 above it.

Dr. Cowan presents us next with Tables of the mortality in the Hospital. The *rate* of mortality is calculated with the exclusion of those dying within 24 hours after admission, these latter being seldom seen by him. This must be borne in mind. We pass over the Tables and quote the summary of results.

The first point that attracts our attention is the relative mortality of the two sexes, and certainly it is very remarkable. The total mortality of the males is 1 in every $6\frac{121}{163}$, while of the females it is only 1 in every $11\frac{21}{101}$.

In the males the mortality is 14.83 per cent.
In the females .. .. .. 8.92 ..

The deaths of the males within the first 24 hours amount to 17.
The deaths of the females .. .. .. .. .. .. .. .. 9.

At almost every period of life embraced in the Table the mortality of the males from Fever exceeds that of the females.

At the age of 15 the mortality is very nearly the same in both sexes.

At the age of 30 the mortality of the males is more than double that of the females.

The rate of mortality is greatest in females at the age of 45.

The mortality of the males under 20 years of age, 6.04 per cent.
.. .. .. .. .. females .. .. .. .. .. 4.90 ..

The total mortality under 30 years of age, 8.35 per cent.
.. .. .. .. .. above 30 .. .. .. 24.84 ..

From the Table of Mortality without reference to sex, and which is a combination of the first two Tables, it appears that, after the age of 10, the mortality from fever slowly increases till the age of 35. From the mortality being 2.63 per cent. at 10 years of age, it has gradually risen to 14.92 at 35 : at 40 it is 22.36, and at 50, 39.06.

III. The next point which engages the attention is the presence or absence of eruption in fever cases.

"Fever may occur without the presence of any eruption during the whole of its progress, or it may be attended by eruptions of various kinds, both when prevailing sporadically and epidemically. The eruptions most commonly attending the fever of this country are petechiæ and vibices—appearing towards the last stage, and symptomatic of a putrid state of the system—an exanthematous eruption, denominated by French writers 'eruption typhoide,' and sudamina, with others of less frequent occurrence. These eruptions may occur singly or in combination.

In many of the epidemic fevers which have taken place, the occurrence of any eruption has not been so general as to form a characteristic feature of the disease, while in others it has been so frequent as to entitle the epidemic to be ranked as an exanthematous, or eruptive fever.

In the epidemic fever of 1816-17 and 18, the fever in the Glasgow Hospital was distinguished, in the worst cases, and in the more advanced stages, by petechiæ and vibices, and was not attended by any exanthematous eruption. In the existing epidemic fever, an exanthematous eruption is present in a vast majority of the patients admitted.

This eruption generally makes its appearance from the fourth to the ninth day of the disease, occasionally, according to my own observations, and those of Chomel, appearing at a later period."

Dr. Cowan proceeds to tabulate methodically the cases of eruption. We need not follow him. He concludes that he is warranted in the inference that the exanthematous eruption is not an essential character of the fever of this country, as during the first six months it occurred in only 49 per cent. of the females, and 63 per cent. of the males ; and, besides this, even in an epidemic, in which it is a distinguishing feature, it is not invariably present, as during the last six months it was absent in nearly 1-5th of those admitted.

Dr. Cowan is of opinion that fever is contagious.

"All the gentlemen who have acted as Clerks in the Fever Hospital for many years past have been attacked with fever, unless they had it previously to their election. During last year twenty-seven of the nurses of the establishment were seized with fever, and five of them died. Several of the students have been affected. One gentleman, who acted as apothecary, died in the House ; and if

I have escaped, it must be attributed either to being past the period of life at which fever usually takes place, or to my being secured by having had two dangerous attacks at an earlier period of my career, when acting as physician's clerk in the Infirmary during the epidemic of 1816-17 and 18."

So far we have traced fever in Glasgow up to the termination of 1836, a period, not of distress, but of unexampled prosperity. From the close of 1836, one of those periodical depressions in trade, arising from the state of our monetary system, has deprived a large portion of the population of the means of subsistence. The pressure of distress has been aggravated by the " strikes" among the workmen. By the strike of the Cotton Spinners on the 8th April, 1837, nearly 8000 individuals, chiefly females for whom no provision whatever existed, were thrown out of employment. To crown all, the average prices of grain were much higher during 1837 than they had been for some time previously.

Much was done by the inhabitants to mitigate these evils: funds were liberally supplied by public contribution, employment was procured for 3,072 males during the months of May, June, and July; soup kitchens were established, from which about 18,500 individuals were daily supplied with food; but, notwithstanding all these exertions, famine and pestilence prevailed to a fearful extent, and the rate of mortality (exclusive of the still-born) rose to 1 in 24.63 of the population. Fever increased as might easily have been anticipated. It extended its ravages over 1-11th of the inhabitants, but its victims were almost exclusively the labouring classes.

| | |
|---|---|
| The number of fever patients treated in hospital in 1837, was | 5387 |
| .. ... .. .. .. .. in 1836, .. | 3125 |
| Being an increase in 1837 of .. . .. | 2262 |
| The number of fever patients treated by the District Surgeons, in their own houses, in 1837, was .. .. .. .. | 2320 |
| .. .. in 1836 .. .. .. .. .. | 716 |
| Being an increase of .. .. .. .. .. | 1604 |

The hospitals were so crowded that many applicants were necessarily rejected. The preceding numbers, therefore, are imperfect.

A reference to the mortality bills will express more forcibly the comparative frequency and mortality of fever during the last three years, than any statement deduced from the hospital returns.

TABLE of the Deaths from Fever recorded in the years 1835, 1836, and 1837.

| | | | | |
|---|---|---|---|---|
| 1835 | 412 | being 1 to every 15.571 of the whole deaths, and 1 to every | 570.388 | of the population. |
| 1836 | 841 | .. .. 10.036 .. .. .. .. .. .. | 290.130 | .. .. |
| 1837 | 2180 | .. .. 5.711 .. .. .. .. .. .. | 116.055 | .. .. |

"The rate of mortality from fever during 1837 has been calculated as high as 1 in 10 of those affected; and upon this supposition 21,800 individuals have been attacked with the disease. The usual average mortality from fever treated in hospitals, is 1 in 15: but the intensity of the epidemic, arising from the poverty and destitution of the population, carried the rate of mortality higher than ever before known in Glasgow.

Upon the assumption that the rate of mortality from fever was 1 in 15 of those attacked in 1835, 1 in 12 in 1836, and 1 in 10 in 1837, which calculations will be found nearly correct, the number of individuals who have been affected with fever in Glasgow during the last three years will be as follows:—

| | |
|---|---|
| In 1835 .. .. .. .. .. | 6,180 |
| 1836 .. .. .. .. .. | 10,092 |
| 1837 .. .. .. .. .. | 21,800 |
| | 38,072 |

The mind cannot contemplate without horror the amount of human misery which the above statement so forcibly expresses."

After presenting a Table of Monthly Deaths from Fever, Dr. Cowan remarks: "Many interesting observations may be drawn from this Table. It shows the slow progress of an epidemic disease when trade is prosperous, compared with what occurs in seasons of distress. Up to November 1836, the period at which the commercial embarrassments were felt, the mortality from fever had not been rapidly increasing. In November it was just about double what it had been in January preceding, the number of deaths being 45 in January, and 89 in November.

The moment, however, the effects of the stagnation in trade extended to the working classes, the mortality increased with fearful rapidity, aided no doubt by the season of the year, the high price of grain, and the scarcity or high price of fuel. The deaths from fever in the four months preceding 1st December, 1836, were 316; for the four months following, 696.

The Table also marks the period at which the epidemic reached its maximum amount of mortality, viz. in the second quarter of 1837, and in the month of May in that quarter, being the month succeeding that in which the strike of the cotton spinners took place, by which 8000 individuals were thrown out of employment."

The labouring classes should not lightly hazard a "strike." The consequences, both physical and moral, are formidable to themselves and their families. Famine, disease, and vice are in its train.

The mortality of males was greater than that of females at every period of life.

The last portion of the Pamphlet before us is occupied with—Remarks suggested by the Mortality Bills.

The Mortality Bill of 1837 exhibits a rate of mortality inferring an intensity of misery and suffering unequalled in Britain, and not surpassed in any city that we are acquainted with on the continent of Europe. The rate of mortality in 1832, during the prevalence of cholera, was 1 in 21.67; but owing to the shorter duration of cholera, less misery and pauperism was produced by it than by fever.

Dr. Cowan thinks he has proved that, during the prevalence of poverty and epidemic disease, the number of marriages and births are uniformly diminished, while, at the same time, the deaths are increased. We find that, in Glasgow—

| | |
|---|---|
| The marriages in 1836 were .............. | 2375 |
| — — 1837, .............. | 2095 |
| Being a decrease of .............. | 275 |
| The baptisms in 1836 were .............. | 3325 |
| — — 1837, .............. | 3085 |
| Being a decrease of ............. | 243 |

The above numbers show the decrease in the *registered* marriages and baptisms only, and not in the total marriages and births, the number of which cannot be obtained. As less than one-half of the births are registered, and as 86 fewer still-born were interred in 1837, the diminution in the number of births may be assumed as about 572.

From further tables furnished by our author, he concludes that, during the last three years, the rate of mortality has been increasing; they likewise demonstrate that the deaths at different periods of life have been fluctuating, and also that, during 1837, the increased mortality has arisen among the adult and not among the infantile population,—a point of much importance, as the mortality of the adult and productive portion of the community occasions much more misery, suffering and pauperism, than an equal or even much greater mortality does at an earlier period of life.

It is in this particular that the increased rate of mortality in 1837 stands prominent above all former years, with the exception of 1832, when the city suffered from the ravages of cholera. In 1832 the per centage of burials under 10 years of age was 42.23, and in 1837, 45.17 in both years, being less than in any year since registers were kept.

The other epidemics, besides fever, which have contributed to swell the lists of mortality during the last three years, have been small-pox, measles, scarlet fever, hooping-cough, and catarrh or influenza; the four first diseases affecting chiefly the infantile portion of the community.

Dr. Cowan sums up thus :—

"It has been proved by the preceding Tables and remarks, that the increase of mortality in Glasgow, during 1835-36-37, has been occasioned by the prevalence of scarlet fever, measles, small-pox, hooping-cough, fever, and influenza, aided in their operation during 1837, by want and destitution among a large body of the population.

The first four diseases were most fatal in 1835-36, and confined their ravages to children under five years of age. Fever prevailed during the whole three years, but its ravages were but slightly felt till 1836 and 1837. In 1835 the deaths from fever, as already stated, were to the total deaths as 1 to 15.57, in 1837, 1 in 4.71. The influenza prevailed chiefly in January, 1837, and to its effects on the extremes of life, and on those labouring under chronic disease, must be attributed a large share of the mortality of the year."

We are happy to learn, that fever is not now desolating Glasgow as our readers will perceive it has done. But as pestilence, by carrying off the weak, breeds healthy seasons to come, these in their turn are nurturing the seeds of future pestilence. Our enormous and increasing manufacturing population is a source of anxiety in more ways than one. It behoves the supreme and the municipal authorities to use all possible efforts to ameliorate the condition, and improve both the physique and the morale of the labouring classes.

---

Elements of Physiology. By *J. Müller*, M.D. &c. Translated from the German, with Notes, by *William Baly*, M.D. Graduate of the University of Berlin. Illustrated with Steel Plates, and numerous Wood Engravings. Part IV. containing Ciliary Motion, Muscular and Allied Motion, Voice and Speech. Price 4s.

We are glad to perceive that this valuable translation is proceeding to its termination. The publication of Müller's physiology is calculated to be of essential service in this country. Our systematic treatises have been hitherto quite unworthy of the state of science, and although we have had individual observations and researches on particular points which displayed a high order of merit, we have had no work which was comparable to the present.

We have before observed that Müller's physiology holds a middle place between the reveries of transcendentalism and the vague superficiality of the dilettante school. Take Burdach's physiology upon the one hand or Mayo's on the other, and we have specimens of both extremes. In the one, many subjects of great importance are barely glanced at or perhaps not mentioned—in the other, every thing, great and small, important and insignificant, is smothered beneath interminable disquisition. There are pages, we might almost say chapters, in Burdach, of such insufferable twaddle, as one could hardly suppose a sane man would indite.

We have intended and we still intend to present some articles to our readers, for the purpose of exhibiting the actual state of physiology. A practical Journal should not neglect, nor should practical men, its readers, undervalue the advances which are made in our knowledge of the functions. A genuine practical man is no blind empiric, no dogged routinist—he is one who is able to *apply* science to the business of life, and to useful purposes. To apply science he should be well acquainted with it. Too many men call and consider themselves practical, simply because they have acquired and employ some empirical experience, not regulated nor directed by high nor comprehensive views.

The Part before us is an extremely instructive one. We have not space at present to redeem our promise, nor to offer a sketch of any topic of importance. We feel inclined to pick out the opinions of Müller on the cure of stammering and his brief hints for its management.

"The method proposed by Dr. Arnott for the cure of stammering, whatever be the result of its practice, is, at all events, founded on a sound physiological view of the nature of the affection. 'Had the edges of the glottis,' says Dr. Arnott, 'been visible, like the external lips of the mouth, the nature of stuttering would not so long have remained a mystery.' The glottis is repeatedly closed in persons who stammer, and the cure of the affection must therefore be effected by conquering this morbid tendency to closure by voluntarily keeping it open as much as possible. For this purpose Dr. Arnott advises that the patient should connect all his words by an intonation of the voice continued between the different words, as is done by persons who speak with hesitation. This plan may afford some benefit, but cannot do everything; since the main impediment occurs in the middle of words themselves, and depends on the abnormal association of the movement of the larynx with certain movements of articulation. Were I called upon to advise a method of treatment in a case of stammering, I would recommend, in addition to Dr. Arnott's plan, the following procedure. I would let the patient practise himself in reading sentences in which all letters which cannot be pronounced with a vocal sound, namely the explosive consonants *b, d, g, p, t,* and *k,* were omitted, and only those consonants included which are susceptible of an accompanying intonation of the voice; and I would direct that all these letters should be pronounced with such a sound of the voice and that their sound should be very much prolonged. By this means a mode of pronunciation would be attained in which the articulation would be constantly combined with vocalisation, and the glottis consequently never closed. When the stammerer had long practised himself in keeping the glottis open without intermission, even between the words by Dr. Arnott's method, and in maintaining the glottis open during and after the pronunciation of every consonant capable of vocalisation and of the vowels, he might proceed to the mute and continuous consonant *h,* and the explosive sounds *g, d, b, k. t, p.* In such a plan of treatment the patient himself would perceive the principle; while the ordinary method—that of Mad. Leigh—is mere groping in the dark, neither teacher nor pupil knowing the principle of the procedures.

There is a kind of defect of speech essentially different from stammering, consisting in a protracted intonation of the voice between words, or the introduction of a more or less prolonged *a* or *au,*—nasal vowel sounds, or peculiar vocal sounds modified by a jingling character between the words, which themselves are correctly pronounced; for example. I . . . a . . . have. It is like the prolonged vibration of a musical instrument beyond the required duration. These sounds form and facilitate the transition from one word to another, and they may frequently be produced as a means of transition; although they, in many instances, also arise from hesitation and want of readiness of the ideas. This mode of speaking sometimes attends stammering, probably because the impediment to the commencement of the next word is avoided by this transition of sounds."

We again recommend the work to the profession and more especially to pupils. To our own we have freely introduced it.

Illustrations of Cutaneous Disease. A Series of Delineations of the Affections of the Skin in their more interesting and frequent Forms; with a Practical Summary of their Symttoms, Diagnosis, and Treatment, including appropriate Formulæ. By *Robert Willis*, Licentiate of the Royal College of Physicians, Physician to the Royal Infirmary for Children, Author of an English Version of Rayer on the Diseases of the Skin, &c. The Drawings after Nature, and Lithographed by Arch. Henning. Fasciculi I. II. III. for January, February, and March.

Dr. Willis is already known, highly favourably, to our readers, as the Translator of the work of M. Rayer on Diseases of the Skin, and as the author of a Treatise on Diseases of the Urinary Organs.

His object in publishing the present Illustrations may be best stated in his own words. These express such just views and explain so clearly what should be the object of all who endeavour to familiarize the profession with the appearances and the management of cutaneous complaints, that we cannot forbear quoting them.

"These diseases are so numerous and so varied in their appearance, and have been designated by such a multiplicity of names, that it is only since our distinguished countryman Dr. Willan thought of attaching Figures to his descriptions that a knowledge of their forms can be said to have been made attainable, or a determinate nomenclature of their genera and species rendered possible. Had the great man I have named but lived to complete his task, it is likely that little would have been left for others to accomplish. His Work, 'On Cutaneous Diseases,' was worthy to have served as a model in every part, for those who came after him; and had it done so, a knowledge of the affections of the skin would now have been less rare than it still undoubtedly remains. But he died in the middle of his career, and the labour of carrying out his plans devolved upon others, who, in my humble apprehension, seem not to have duly appreciated the end he had in view. This, as I conceive it, was to render an account of the nature, symptoms, and diagnostic marks of the disease generally of which his Figure was the individual expression, and to apply the knowledge thus acquired to its legitimate end, the alleviation or cure of the malady. Dr. Willan gave Plates, mostly embracing single subjects, which he illustrated with a text at once the most elegant, the most learned, and the most practical that can be imagined.

The serious expense of a work on the plan of that of Willan, occasioned principally by the great cost of copper-plate engraving, was, however, an insurmountable obstacle to its general diffusion, and therefore to its usefulness. Other branches of natural knowledge have the titled and the wealthy for their patrons and cultivators; our honourable profession is not adopted either by the rich or the great; the objects it pursues are not held of general interest, and he who publishes expensive plates on subjects of medical science, has generally had to bear the cost of them himself. This circumstance led to the adoption of a procedure which, in regard to the Diseases of the Skin, I cannot but view as fraught with something like a necessity of failure in the end to be obtained. This was the plan of giving miniature representations of Cutaneous Diseases, upon square patches of integument, and setting a multitude of these microscopic pictures within the frame of a single page. By this device the eye is so much distracted, that what is sought for is almost certain to elude its search.

Since I translated the excellent work of Dr. Rayer, which was begun in the year 1833, I have paid much attention to the subject of dermal pathology; and in the art of Printing from Stone, I have seen a means of realizing the objects which I imagine ought to be kept in view in every Iconographic work—the union of pictorial representation with practical knowlege at a moderate expense. For some considerable time I have, therefore, engaged an artist to make drawings for me of those forms of cutaneous disease that struck me as most interesting, which occurred either in the course of my own practice, (especially at the Royal Infirmary for Children, where the opportunities of observing the diseases of the skin in childhood are all but unlimited,) or that were kindly recommended to my notice by those among my friends who knew the interest I took in the subject. Determined to avoid everything like crowding my plates, I have resolved rather to content myself with giving delineations of eighty or ninety subjects that shall never afterwards be confounded with one another, nor with anything else, than to produce three or four hundred, among which uncertainty and confusion must of necessity have remained. At the same time I see that three or four hundred figures are many more than are required to convey a very perfect knowledge of the subject. The number of varieties is, indeed, endless—that of species, to which varieties are easily referable, is much more limited. On from eighty to one hundred plates, embracing the same number of subjects, I conceive that I can exhibit almost every disease that commonly occurs or that is really important.

Nor could I, with my views, be satisfied to give a barren account, two or three lines in length, of the individual instance I have represented. This were but playing the showman to the painter. I have confronted each plate with an account, in as brief terms as possible, of every particular of greatest importance in the Symptoms, Diagnosis, Prognosis, and Treatment of the disease figured; by which I trust that my work, when finished, will be found not only a Connected Series of Representations of the Diseases of the Skin, but a Compendious Practical Guide to a Knowledge of their intimate Nature, and of the Means of Treating them successfully."

The first Fasciculus presents Delineations of Lepra Papyracea (too lilac-like in colour)—of Herpes Zoster—of Intertrigo cum Vesiculis (not bad)—and of Lupus non Exedens. The artist has throughout thrown too much of a purplish hue upon his subjects.

In the second Fasciculus we have Lepra Vulgaris (still the lilac predominates)—Pompholyx—Purpura in the shapes of Ecchymoses and Petechiæ.

The third Fasciculus presents us with representations of Trichosis (Porrigo) Scutulata—of Eczema Capillitii—of Ecthyma Capillitii—and of Pityriasis Capitis.

If we might hint a fault, it is in the colouring. That is not yet, we think, perfect. No doubt it may and it will be improved. The execution of the work is in all other respects excellent. It is likely to answer the intentions of the author, and is eminently adapted to diffuse a knowledge of the various forms of cutaneous affections. To the country practitioner it will prove of great service, and we cordially recommend all our readers to patronise it, both for the sake of the author and themselves.

---

ILLUSTRATIONS OF OSTEOLOGY. By *Theodore G. Boisragon*, M.D. Cheltenham.

The elder Dr. Boisragon is well known as a very excellent and eminent physician of Cheltenham. The son will, we trust, tread in the father's steps. The present is an earnest of his zeal and industry. The Illustrations before us are beautifully executed, judiciously selected, and well adapted to convey, in the absence of the bones themselves, an accurate notion of their points of demonstration.

THE SURGEON'S VADE MECUM, A HANDBOOK OF THE PRINCIPLES AND PRACTICE OF SURGERY. Illustrated with numerous Wood Engravings. By *Robert Druitt*, M.R.C.S. London, Renshaw, 1839.

If we know anything of the wants and tastes of students, this Vade Mecum will *take*. It gives them what they need, concise descriptions and pat directions. Nor are the omissions considerable. Such a book was a desideratum, and, if we are not mistaken, Mr. Druitt has made a *hit*.

---

### NATURAL HISTORY.

I. A HISTORY OF BRITISH BIRDS. By *William Yarrell*, F.L.S. V.P.Z.S. Illustrated by a Woodcut of each Species and Numerous Vignettes. Van Voorst. Part XI., completing the First Volume.

This agreeable work issues regularly from the press. It is one which should be on the drawing room, as well as on the library table—an interesting present to the young, a pleasant companion for the old. The present Fasciculus keeps the promise made by its predecessors.

II. A HISTORY OF BRITISH REPTILES. By *Thomas Bell*, F.R.S. F.L.S. Professor of Zoology in King's College. Illustrated by a Woodcut of each Species, with some of the Varieties, and numerous Vignettes.

It is not necessary to introduce Mr. Bell, or his works, to our readers. He is excellently qualified for an undertaking of the kind before us, and a very deserving one it is. We extract the following account of the method of using the poison apparatus in serpents.

"When the animal inflicts the wound, the pressure on the tooth forces a small drop of the poison through the tube; it passes through the external orifice, which is situated on the concave side of the curved tooth, and is in the form of a slit. The manner in which the blow is inflicted is as follows. The animal generally throws itself in the first place into a coil more or less close, and the anterior part of the body is raised. The neck is bent somewhat abruptly backwards, and the head fixed almost horizontally. In an instant the head is, as it were, launched by a sudden effort towards the object of its anger, and the erected tooth struck into it, and withdrawn with the velocity of thought. It is found by experiment that the effect of subsequent wounds is greatly diminished either by the diminution of the quantity of venom, or by some deterioration of its strength; so that if a venomous Serpent be made repeatedly to inflict wounds, without allowing sufficiently long intervals for it to recover its powers, each successive bite becomes less and less effective. A gentleman of my acquaintance had some years since received a living Rattlesnake from America. Intending to try the effects of its bite upon some rats, he introduced one of these animals into the cage with the Serpent; it immediately struck the rat, which died in two minutes. Another rat was then placed in the cage; it ran to the part of the cage farthest from the Serpent, uttering cries of distress. The Snake did not immediately attack it; but after about half an hour, and on being irritated, it struck the rat, which did not exhibit any symptoms of being poisoned for several minutes, and died at twenty minutes after the bite. A third, and remarkably large rat, was then introduced into the cage. It exhibited no signs of terror at its dangerous companion, which, on its part, appeared to take no notice of the rat. After watching for the rest of the evening, my friend retired, leaving the serpent and the rat together; and on rising early the next morning to ascertain the fate of his two heterogeneous prisoners, he found the Snake dead, and the muscular part of its back eaten by the rat. I do not remember at what time of the year this circumstance took place, but I believe it was not during very hot weather."

We cordially recommend this work also to our readers. We shall take care to place it in our own library.

III. THE NATURALIST'S LIBRARY. Conducted by Sir *William Jardine*, Bart. F.R.S.E., F.L.S.. &c. Mammalia, vol. VIII. Amphibious Carnivora, including the Walrus and Seals, also of the Herbivorous Cetacea, &c. By *Robert Hamilton*, Esq., M.D., F.R.S.E., M.W.S., &c. S. Highley, London, 1839.

We take shame to ourselves for not having directed the attention of our readers who love natural history (and who do not) to the Naturalist's Library. It is a very delightful work, and should grace the shelves of any man who has a family. Productions of this kind will, we hope, displace the trash which was formerly in the hands and on the lips of young persons, and give them that relish for the study of nature, so invigorating to the mind, and so calculated to fit it for the sober occupations of life.

In the advertisement to the present volume we observe it stated, that:—

"Amongst the various benefits which the volumes of the Naturalist's Library have conferred upon the study of Natural Science, not the least valuable has been the publication of groupes, or families, of animated beings, of the extent of which we know that the public in general had no previous conception.

Such is our present volume on the history of the Amphibious Carnivora, in which are described all the known species, illustrated by numerous plates and wood-cuts, and these interesting details congregated together at the very trifling expense of *six shillings*."

Independently of wood-cuts this volume contains thirty well-executed coloured plates, and not the least amusing are three of the "Great Sea Serpent," and the "Craken." We cordially recommend the work to our readers, and we shall not fail to notice future volumes as they appear.

IV. A GENERAL OUTLINE OF THE ANIMAL KINGDOM. By *T. Rymer Jones*, F.Z.S. Professor of Comparative Anatomy in King's College, London. Parts III., IV. and V. Price 2s. 6d. each.

We noticed in our last the commencent of this useful work. We have now to chronicle its continuation.

The Second Part almost completed the First Division of the Animal Kingdom—the ACRITA, comprising:—

1. Sponges.
2. Polyps.
3. Polygastric Animalcules.
4. Acalephæ.
5. Parenchymatous Entozoa, or Sterelmintha.

With the Third Part we come upon the Second Division, the Nematoneura, comprising:

1. Bryozoa, or Polyps with Ciliated Arms.
2. Rotifera.
3. Epizoa.
4. Cavitary Entozoa, or Cœlelmintha.
5. Echinodermata.

We present the following brief sketch of the division of Nematoneura, the second step in the Radiata of Cuvier. It is contained in the First Part of the Outlines before us.

"The nervous matter is distinctly aggregated into filaments, and in some cases nuclei of neurine, which may be regarded as rudimentary nervous centres, have been noticed. It is to be lamented, however, that in this most interesting group of animals, in which we have the first development of most of the organs subservient to the vital functions, the extreme minuteness of some genera, and the difficulty of distinctly observing the nervous system in the larger species, has prevented our knowledge regarding their organization, in this particular, from being of that satisfactory character which it is to be hoped it will hereafter attain to.

Owing to the want or imperfect condition of the nervous centres, the nematoneura are necessarily incapable of possessing external organs of the higher senses, the general sense of touch being as yet the only one of which they are indubitably possessed; yet in their muscular system they are much more efficiently provided than the acrite orders, as the development of nervous threads of communication renders an association of muscular actions possible; and therefore, co-apparent with nervous filaments, we distinguish in the structure of the nematoneura distinct fasciculi of muscular fibre, and powers of locomotion of a much more perfect description.

The digestive apparatus is no longer composed of canals merely excavated in the parenchyma of the body, but is provided with distinct muscular and membranous walls, and loosely attached in an abdominal cavity.

The circulation of the nutritious fluid is likewise carried on in a separate system of vessels, distinct from the alimentary apparatus, yet still unprovided with a heart, or exhibiting pulsations for the forcible impulsion of the contained blood.

The fissiparous mode of reproduction is no longer witnessed, an obvious consequence of the increased complexity of structure, and these animals are for the most part androginous, or capable of producing fertile ova, without the co-operation of two individuals."

The accounts of the Cœlelmintha, the Bryozoa, the Rotifera, the Epizoa, and the Echinodermata, are concise, clear, and interesting. The woodcuts illustrate the text, and both are executed in the most creditable manner.

In Part IV. we are introduced to the Homogangliata, the Articulata of Cuvier, the third grand division of the Family of Nature.

It includes, writes Mr. Jones, an immense number of living beings adapted by their conformation to exist under a far greater variety of circumstances than any which we have hitherto had an opportunity of examining. The feeble gelatinous bodies of the Acrita are obviously only adapted to an aquatic life; and accordingly they are invariably found either to inhabit the waters around us, or to be immersed in the juices of living animals upon which they subsist. The Nematoneura, likewise, are all of them too imperfect in their construction to admit of their enjoying a terrestrial existence, for, possessing no nervous centres adequate to give force and precision to their movements, they are utterly incapable of possessing external limbs endowed with sufficient power and activity to be efficient agents in ensuring progression upon land; neither are any of them furnished with those organs of sense which would be indispensable for the security of creatures exposed to those innumerable accidents to which the inhabitants of a rarer element are perpetually obnoxious: the NEMATONEURA therefore are, from their organization, necessarily confined to a watery medium.

But the type of structure met with in the HOMOGANGLIATA admits of far higher attributes, and allows the enjoyment of a more extended sphere of existence: senses become developed proportionate to the increased perfection of the animal; limbs are provided endowed with strength and energy commensurate with the development of the nervous ganglia which direct and control their movements; and instincts are manifested in relation with the increased capabilities and more exalted powers of the various classes as they gradually rise above each other in the scale of animal development.

The most obvious, though not the most constant, character which distinguishes the creatures we are now about to describe, is met with in their external conformation; they are all of them composed of a succession of rings formed by the skin or outward integument, which from its hardness constitutes a kind of external skeleton, supporting the body, and giving insertion to the muscles provided for the movements of the animal. In the class CIRRHOPODA alone is this external characteristic wanting, and the Homogangliate organization masked by a tegumentary testaceous coat of mail, which they seem to have borrowed from the molluscous type.

This division includes:—1. Cirripeda. 4. Insecta.
2. Annelida. 5. Arachnida.
3. Myriapoda. 6. Crustacea.

The properties and habitudes of the Annelida, Myriapoda, and Insecta occupy Parts IV. and V., and of the mode in which they are treated we can speak as favourably as we have of the preceding Parts. Altogether the work of Mr. Jones is a highly meritorious one, and should be in the possession of every medical man who wishes to keep pace with the current knowledge in the department of Zoology.

---

ELEMENTS OF CHEMISTRY, INCLUDING THE RECENT DISCOVERIES AND DOCTRINES OF THE SCIENCE. By the late *Edward Turner*, M.D. Sixth Edition, enlarged and revised. By *Justus Liebig*, Ph.D. Professor of Chemistry in the University of Giessen; and *Wilton G. Turner*, Ph.D. ORGANIC CHEMISTRY by Professor Liebig. Part III. No. 1. Price 3s. 6d. London. Taylor and Walton. 1839.

We have watched with interest the appearance of the successive Parts of this Edition of the late Dr. Turner's Chemistry. We have recommended and we recommend it as the most valuable work of the kind in our language. The present Part, devoted to Organic Chemistry, opens up quite a new field in that region of chemical science. It would be impossible to present an intelligible sketch of it in any moderate compass, and we must therefore content ourselves with recommending the original to the profession in the very strongest terms.

---

### The Quarantine Laws. Mr. Holroyd's Letter to Sir J. C. Hobhouse on the Abuses and Inconsistencies of the said Laws. 1839.

We suspect that the fate of the quarantine laws will not be very unlike that of the corn-laws. Neither of them may be entirely abrogated, nor immediately; but both of them are destined to considerable modification. The mass of absurdities, inconsistencies, and diableries exposed in this letter, would fill a whole article, and we cannot condense it. The following extract from a letter of Dr. Gregson to Mr. Holroyd will give the reader some idea how the plague doctors manage these things in the *East*.

"On arriving at Beyrout I was put in Quarantine: during this time the Lazzaret and town were alarmed by the Doctor of the Commission reporting a Greek sailor from Cyprus attacked by plague; luckily the Commission consisted of the Governor, who was a well educated Turk, a paid Inspector, and the Doctor. This case caused a sensation, as the trade with Cyprus is great. The Governor called in another doctor, who said it was not plague. During this perplexity the Governor, hearing I was in the Lazzaret (he had known me well in Egypt), sent for me to have my opinion; I found the patient suffering from an extensive gangrene with sloughing, caused by a severe contusion, produced by a blow from the cable breaking when they were heaving it up. Astonished at the ignorance, or rather malicious conduct of the Doctor, (he had an interest, in being paid so much for visiting people from infected parts) I gave my opinion in reprobation of the Doctor's conduct; he was immediately discharged. Had he belonged to the Alexandrian Commission, it would have screened him and persecuted those who differed from him. Our Consul Mr. Thurburn is an honourable exception; he did not belong to it, and has interfered to prevent British subjects from being dragged to the Lazzaret. In Alexandria I have been sent by order to inspect various cases in the Lazzaret. The Commission Doctors got on these occasions two Doctors to side with them; for these services one is now Commissioner Doctor at Alexandria, the other so at Damietta, viz. Couloutchi and Reggio."

Yes, Yes! we shall never want the plague of quarantines, while we have such purveying doctors as the above, who can convert the blow of a cable into a brace of buboes! The tenor of the various answers to various questions collected by Mr. Holroyd is all to corroborate the opinion that the plague is an endemic frequently spread out or elevated into an epidemic, and that it is caused by a febrific miasm, but occasionally propagated by emanations from the bodies of infected persons, as is the case with all, or almost all fevers.

---

### The Medical Portrait Gallery.

Twelve Parts of this amusing and instructive work have now been published. They contain memoirs, more or less complete, of many of the eminent men of our profession. If the work is patronised, as it ought to be, it will be really, what its name implies, a gallery of portraits of whatever has been most learned, and famous amongst us.

Such a gallery is wanted. Success is more the test of merit, of one description or another, than the unsuccessful are willing to allow. In our profession, great success has seldom been attained without corresponding exertions or without the possession of some distinctive quality of mind. Success too, with us, almost certainly indicates no wide departure from moral rectitude. A history of medical success becomes a lesson for the future candidate. He reads, in the golden page, the bright results of industry, genius, and worth, and while he reads, the glowing sentiment of admiration insensibly blazes into the desire to emulate.

There is a sacred pleasure too in contemplating the images of men who have dignified the sciences we profess, who, in fact, have made it the science that it is. While we gaze we resuscitate buried generations—study physic from the votive tablets in the temples of the Greek—pace the cloistered walks of Oxford with Linacre—enjoy the hospitality and the learning of Mead—or roar at the jest of honest Abernethy.

We cannot of course give a formal account of each distinguished person whose portrait and memoir grace the Gallery of our excellent friend, Mr. Pettigrew. But we think that a few anecdotes, some collectanea, from his pages may not be unacceptable.

1. *The Death of Vesalius.*—The following account, which has been much distorted by tradition, is contained in a letter from Hubert Languet to Gaspar Peucer.

" Vesalius (he says,) believing a young Spanish nobleman, whom he had attended, to be dead, obtained leave of his parents to open him, for the sake of inquiring into the real cause of his illness, which he had not rightly comprehended. This was granted ; but he had no sooner made an incision into the body, than he perceived the symptoms of life, for, opening the breast, he saw the heart beat. The parents, coming afterwards to the knowledge of this, were not satisfied with prosecuting him for murder, but accused him of impiety to the inquisition, in hopes that he would be punished with greater rigour by the judges of that tribunal, than by those of the common law. But the king of Spain interposed, and saved him ; on condition, however, that, by way of atoning for the crime, he should undertake a pilgrimage to the Holy Land."

Boerhave and Albinus say that he was condemned by the Inquisition, from which he was, by the influence of Philip, saved. He made the pilgrimage with James Malatesta, general of the Venetian army, whom he accompanied to Cyprus, whence he passed to Jerusalem. There is much in the account given to excite unbelief as to its credibility, from the extent to which dissection must necessarily be made before the heart could be exposed ; yet the possibility of the muscular fibres of this organ acting by their principle of irritability, a principle unknown in the time of Vesalius, remaining even after vitality had quitted the body, may tend to sanction the statement made.

In 1563, the principal chair at Padua became vacant by the death of his pupil, Fallopius, and Vesalius was, at the invitation of the senate of Venice, induced to return to succeed this celebrated physician. On his voyage, however, a a storm arose—he was shipwrecked—thrown upon the Island of Zante, and there perished of hunger, on October 15, 1564. His body was recognised by a goldsmith of Venice, who procured an honourable entombment for it, in the church of St. Mary, of that island, and he placed the following inscription over his grave :—

ANDREÆ VESALII BRUXELLENSIS TUMULUS.

QUI OBIIT IDIBUS OCTOBRIS,

ANNO MDLXIV.

ÆTATIS VERO SUÆ QUINQUAGESIMO,

QUUM HIEROSOLYMIS REDIISSET."

2. *Abernethiana.*—A man of rank consulted Mr. Abernethy, and was received by him with remarkable rudeness. Upon some severe remark being made, the patient lost his temper and told Mr. A. he would make him *eat his words.* " It will be of no use," said Mr. A., coolly, "for they will be sure to come up again !"

" Pray Mr. Abernethy, what is a cure for gout ?" was the question of an indolent and luxurious citizen. " Live upon sixpence a day—and earn it," was the cogent reply.

He is reported to have been consulted by the late Duke of York; and he stood before his royal highness, whistling, with his hands in his breeches-pockets, as usual. The duke, astonished at this conduct, said, "I suppose you know who I am:" "Suppose I do," said he, "what of that?" And his advice to his royal highness was given thus: "Cut off the *supplies*, as the Duke of Wellington did in his campaigns, and the enemy will leave the citadel."

A barrister had a small ulcer on the leg which was difficult to heal, and he determined to apply to Mr. Abernethy. Aware of his impatience and eccentricity, he, immediately upon entering his room began to pull down his stocking. "Holloa! holloa! what the devil are you at?" said the surgeon. "I don't want to see your leg; that will do—put it up, put it up," The patient did so; but justly dissatisfied with the imperfect manner in which his case had been considered, he, instead of the usual fee, placed a shilling only upon the table. "What is this?" said Mr. A. "Oh," replied the barrister, "that will do—put it up, put it up," and coolly walked away.

*Abernethy as a Lecturer.*—The lecture-room was the grand theatre upon which Mr. Abernethy displayed; there, indeed, he "shone eccentric like a comet's blaze!" and there he would indulge his disposition and propensities to an extent which occasioned the pupils frequently to regard it as an exhibition, and call it an "Abernethy at Home." His mode of entering the lecture-room was often irresistibly droll—his hands buried deep in his breeches-pockets, his body bent slouchingly forward, blowing or whistling, his eyes twinkling beneath their arches, and his lower jaw thrown considerably beneath the upper. Then he would cast himself into a chair, swing one of his legs over an arm of it, and commence his lecture in the most *outré* manner. The abruptness, however, never failed to command silence, and rivet attention.

"'The Count was wounded in the arm—the bullet had sunk deep into the flesh—it was, however, extracted—and he is now in a fair way of recovery.' That will do very well for a novel, but it won't do for us, Gentlemen: for 'Sir Ralph Abercromby received a ball in the thick part of his thigh, and it buried itself deep, deep: and it got among important parts, and it couldn't be felt; but the surgeons, nothing daunted, groped, and groped, and groped,———and Sir Ralph died.'"

*Abernethy at the last.*—His eccentricity continued during his existence, and towards the last he is reported to have joked upon the œdematous state of his legs produced by the disturbance of the circulation and his difficulty of breathing. Some one inquired of him how he was? to which he replied, "Why, I am better on my legs than ever: you see how much stouter they are!" His hobby retained full possession also to the end of his life. He attributed his disease to the stomach. He said, "it is all stomach; we use our stomach ill when we are young, and it uses us ill when we are old." But it is not a little singular, that he expressly enjoined that no examination of his body should take place!

3. In Part VII. is an interesting memoir of William Hunter. Perhaps our readers may not be aware that the Doctor proposed to Government building a National Anatomical Museum, a proposition which was virtually rejected.

Dr. Hunter's solicitation was to have the grant of a piece of ground, upon which he might expend six or seven thousand pounds in erecting a building fit for the purpose of anatomical study, and a "*Plan for establishing a museum in London for the improvement of anatomy, surgery, and physic*," was forwarded with the "memorial." Lord Bute recommended the plan to the Right Hon. George Grenville, and Mr. Hawkins presented a memorial to the king. Delay after delay, however ensued, and the proposal fell to the ground. The Earl of

Shelburn felt the utility of the scheme, and proposed that it should be effected by a subscription, and put his own name down for 1000 guineas. Dr. H. rejected this from motives of delicacy, and commenced building a theatre, museum, &c. on a spot of ground in Great Windmill-street, which has ever since borne the name of the Hunterian School.

*Dr. Hunter's Death Bed.*—His composure and resignation at the last deserve to be recorded. Turning to his friend Dr. Combe: "If I had strength enough to hold a pen, (said he,) I would write how easy and pleasant a thing it is to die."

"O, what a wonder seems the fear of death,
Seeing how gladly me all sink to sleep;
Babes, children, youths, and men,
Night following night, for threescore years and ten."

COLERIDGE.

*His Embalming of Mrs. Van Butchell.*—His freedom of speech, and ease in treating his subject, once occasioned him some little trouble. It was customary to deliver, at the conclusion of each course of lectures, a discourse on the mode of making anatomical preparations. This introduced the embalmings of the ancient Egyptians, and Dr. H. alluded to the process of preserving a body from putrefaction as a matter of little or no difficulty, and one that might most easily be accomplished. Among his pupils at this time was a man afterwards much celebrated in London by his singularity of manners and empiricism, the late Martin Van Butchell. Upon the conclusion of the lecture he approached the Doctor, stated the interest he had felt upon the subject of embalming, and was anxious that the Doctor should effect this for him upon the body of his wife, who was at that time lying in the last stage of a consumption. The Doctor had spoken of the matter as being so trifling, that he could not refuse this request, and, upon the death of Mrs. Van Butchell, the operation was effected.

4. *Baillie's Generosity.*—The following instances of the generosity of Dr. Baillie deserve to be recorded. Sir C. Bell has related the first.

"The merest chance (says he) brought me acquainted with a circumstance very honourable to Dr. Baillie. While still a young man, and not affluent, his uncle William, dying, left him the small family estate of Long-Calderwood. We all know of the unhappy misunderstanding that existed between Dr. Hunter and his brother John. Dr. B. felt that he owed this bequest to the partiality of his uncle, and made it over to John Hunter. The latter long refused; but, in the end, the family estate remained the property of the brother, and not of the nephew of Dr. Hunter."

Mr. Wardrop relates several instances of generosity on the part of Baillie.

"A young lady who was suffering severely from a pulmonary complaint, asked his advice, and he recommended her to spend the winter months in a milder part of the country. He found that her circumstances would not admit of her trying this last resource to regain her health, and, to enable her to do so, he instantly gave her an adequate sum of money."

"A lady, whose rank in life was far above her pecuniary resources, had an illness, when his attendance became important, and during which he regularly took his usual fee, until it was no longer necessary; he then left in a bag the whole amount of what he had received, offering to the lady, as an apology, that he knew that, had he once refused to take his fee during his attendance, she would not have permitted him to continue it."

5. *Advantages of Royalty in Death-bed Scenes.*—The following extract from the life of Mr. Wardrop, as compiled by Mr. Pettigrew in a late number of his biography of the living and the dead, will excite some reflections in the minds of our readers, but whether of the joyous or dolorous kind, we shall leave themselves to determine.

"There are circumstances connected with the last illness of George IV. which deserve notice here, as forming part of Mr. W's professional history. About eight weeks previous to the King's death, Mr. Wardrop visited his majesty at Windsor, the King then being considered in a state of convalescence from a a severe inflammatory attack of the chest. From the state of the respiratory and circulating organs, Mr. W. was convinced that the condition of the heart was much altered; and on returning to London, he instantly repaired to Sir Henry Halford, purposely to direct his attention to this important circumstance, and to urge him to visit his Majesty on the following day, which was several days sooner than had been appointed. His Majesty continued to be attended by the late Mr. O'Reilly, on whose practical talents the King had great confidence, as well as by Sir H. Halford; and Mr. Wardrop's visit at Windsor was not repeated until he was called upon by the 'lord in waiting,' commanding him immediately to repair to Windsor. This visit was made on Sunday the 25th of April, 1830. On this occasion, when Mr. W. entered the royal bed-chamber, he found his Majesty alone, sitting upon a couch, his countenance bespeaking some serious mischief. He had great embarrassment in breathing; and, after detailing every circumstance of his case, and Mr. W. had made a most careful examination of the chest, his majesty said, in a firm and decided tone, 'Tell me, my good friend, what you think, really and truly, is the matter with me, for I am confident that there is something much more serious than ——— either thinks or chooses to tell me.' Mr. W. then stated to His Majesty that the difficulty of breathing arose entirely from an impediment of the circulation of the blood through the heart. His Majesty replied, in a manly tone, 'tell me, Wardrop, honestly, if you think I shall recover.' To which Mr. W. answered, that his condition was by no means hopeless, though His Majesty must be perfectly aware that any disease of a vital organ, like the heart, could not be altogether free of danger.

After having remained in the royal bedchamber about forty minutes, Sir William Knighton entered, and Mr. W. retired. The King having stated to Sir William Knighton the opinion which Mr. W. had given of his case, and Mr. W. having repeated that opinion to Sir W. K. at his own particular desire, Sir William then requested Mr. W. to state in writing that opinion, and the treatment he proposed to adopt, in a letter addressed to Sir Henry Halford, and which he would deliver to Sir Henry on his arrival at Windsor in the evening. In this letter, Mr. W. intimates his opinion that the affection of the heart might be dependent on an *arthritic diathesis*, and that, if by pediluvia and the application of stimulants to the legs and feet, the gout could be brought to manifest itself in the limbs, His Majesty might be relieved. He also suggested, that leeches should be applied to the region of the heart.

The *post-mortem* examination of the King demonstrated the morbid condition of the heart, and afforded a most satisfactory proof of the correctness of the opinion Mr. W. had formed during the King's illness, and the propriety of the treatment he had proposed. But after the period of this visit, Mr. W's attendance at Windsor terminated, though he afterwards learnt that the King had repeatedly expressed a wish to see him, and also surprise at his never having returned to the palace. Such, however, was the helpless condition of the monarch on his death-bed, that he was entirely under the control of a few individuals who surrounded his person."

We shall resume on a future occasion our extracts from this interesting work. We trust sincerely that it will be patronised.

### A LETTER TO DR. CHAMBERS ON SEVERAL IMPORTANT POINTS RELATING TO THE NATURE AND TREATMENT OF GOUT. By Sir *Charles Scudamore*, M.D. pp. 59. Longman and Co.

THE days of "flannel and patience" in the cure of gout, have long gone by—nor is the disease so cherished as it was at the beginning of the present century, when Dr. Heberden remarked that—

"People are neither ashamed nor afraid of it; but are rather ambitious of supposing that every complaint arises from a gouty cause, and support themselves with the hope that they shall one day have the gout, and use a variety of means for this purpose, which, happily for them, are generally ineffectual." Again—"For this (the gout) seems to be the favorite disease of the present age in England; wished for by those who have it not, and boasted of by those who fancy they have it; though very sincerely lamented by most who in reality suffer its tyranny."

At present, the dread of *having* the disease is nearly as great as that of *curing* it formerly was. The passive treatment suddenly changed into a very active one, and Dr. Kinglake sent some scores of patients across the river Styx, by way of securing for himself a warmer reception than that which he accorded to gout. Then came the eau medicinale—so magical in its workings that it was considered a boon from the skies. But the blessing was quickly found to be a curse—as is the case with many supposed blessings in this world! The colchicum autumnale succeeded, and though less magical in its effects, has been more safe and beneficial in its operation. It is now, and probably will remain so, the best remedy in the paroxysm of gout—though it cannot be considered at all in the light of a prophylactic. This last and most important measure must be sought in the avoidance of the causes, and correction of *their* effects on the liver, stomach, and intestines.

It is well known that Sir Charles early introduced, and has long clung to a formula of his own—the acetum colchici combined with magnesia and sulphate of magnesia, thus acting both on the kidneys and the bowels. He now uses the acetous extract of colchicum prepared by Mr. Garden, by the evaporation of a saturated infusion of the dried roots in acetic acid, over a warm bath. The formula in the new Pharmacopœia is not exactly according to the directions of Sir Charles. Colchicum, Sir Charles thinks, is still grossly abused in practice, and productive of serious consequences. He employs the remedy only as "a palliative agent for the control of an inconvenient degree of the gouty suffering and irritation—and that in the mildest form of its preparation, and in combination with other remedies." The real curative plan consists in the correction of various functional disorders. In chronic cases he also endeavours to alter the state of the blood itself.

Sir Charles's note-book has furnished him with ample data, he thinks, to estimate the nature of gout. In the first place, he observes, the fit may occur as the effect of repletion, proving a kind of safety-valve in the removal of plethora. Where gout is complicated with, or dependent on other diseases, he thinks the colchicum should rarely be used, as the explosion of gout itself may be useful in arresting the other malady—as for instance apoplexy or tendency to it.

"The brain is sometimes very curiously affected by gouty irritation, not yet developed in its proper form. A gentleman, who had never experienced gout but once, had undergone great fatigue of body, with anxiety of mind. He was seized with inflammation of the calf of the leg, which was considered to be erysipelas; and at the same time his imagination became affected with this extraordinary delusion, that he thought himself a serpent crawling at the bottom of a cauldron. A blister was applied to the neck, and active medicines were administered ; on the following day, acute gout attacked the ball of the great toe, and immediately the senses were restored."

Headaches in early life are sometimes removed by gout in maturity. Every body has seen asthma and gout alternate. Neuralgic affections of the scalp are often relieved by a paroxysm of gout in the feet. Cramps and fidgets are frequently of a gouty nature, and brought to a conclusion by a paroxysm. Gastralgia, palpitation of the heart, giddiness, &c. are not uncommon marks, under which gout harasses its victims for years, and which are relieved, at least for a time, by a regular attack on less vital parts.

"The mind, not less than the body, is brought under the powerful influence of suppressed gout. A gentleman, threatened with gout when beginning a tour in Italy, took colchicum in repeated small doses, and averted the fit; but was rendered so listless and lethargic, 'so truly miserable,' to use his own words, that at the Florentine gallery he could scarcely resist throwing himself down on the floor, instead of admiring the works of art by which he was surrounded. His first wish then was to have the fit which he had been so studious to prevent.

A military man, of the firmest mind when in health, had in the same manner made use of colchicum, and suppressed a coming fit. His nervousness became extreme. He would only see a chosen friend, and on the slightest occasion burst into involuntary tears, without any moral cause.

Nerves are sometimes, in a fit, the only texture apparently affected: the most intensely painful attack which I ever saw, was in the nervous branches distributed over the foot. With the exception of a little fulness of the veins, there was no external appearance of disease. The case was very peculiar ; the gentleman was of full habit ; no relief was afforded by colchicum, or by opiates, until he had been very freely bled from the arm."

Several pages of this letter are occupied by examples of the vagaries which gout plays on the system ; but most of them are familiar to practitioners of any experience.

In respect to the treatment, Sir Charles thinks that the functions of the liver are almost invariably disordered—"often standing in the relation of cause and effect." This, we must confess, is both too broad and too narrow a basis for the pathology of gout. So strong is often the hereditary diathesis, that, in spite of all due attention to the functions of the liver and the whole of the digestive organs, yet gout will occur. Nevertheless we admit that, in a great majority of cases, "it is of primary importance to give some mercurial purgatives and alteratives, for the purpose of evacuating *vitiated secretions*, and inducing more healthy action." But we would ask Sir Charles, or any other practitioner whether this precaution is not necessary in almost every complaint, where there is any suspicion of disordered action or vitiated secretion ? Sir Charles cautions us against salivation, in such cases,—a caution hardly necessary. He prescribes the acetous preparation of colchicum as a valuable palliative—sometimes in a state of effervescence—with Battley's liq. op. sed. as a sedative —sometimes the acetate of morphia, joined with a sudorific.

After the violence of the attack is over, Sir Charles recommends, and we think wisely, *local* remedies—seldom leeches.

"In the advanced stages of the disease, other topical means may be used with great advantage ; as the veratria liniment; linim. sapon. c. with liquor. ammon. acet. and tincture of opium; linim. sapon. c. with linim. camphor. c. and tinctura lyttæ, &c. Finally, benefit will be derived from the daily or occasional sponging with a mixture of tepid salt water and vinegar; friction and shampooing; with bandage also, if the vessels should be so weak as to cause œdema."

From some little acquaintance with gout, both *personally* and professionally, we hesitate not in giving it as our opinion, none of the above applications is superior or even equal to simple spirituous evaporating lotions, kept constantly on the inflamed parts, and applied in a tepid state. Sir Charles appears to be a little stricken with the "*soda-phobia*" of Magendie and his disciples. We are no advocates for the habitual use or abuse of soda, where acidities do not prevail in the stomach; but where they do prevail, we maintain that alkalies are highly beneficial, and extremely soothing. We shall conclude with the following judicious advice.

"Although no truism is more known, it cannot be too often repeated, that almost all persons who have the means, take daily an excess of food beyond what is required and useful.

Every one has too much occasion to say with the poet,

——— video meliora proboque,
Deteriora sequor.

In some instances of dyspepsia, the digestive powers are so weakened, that the conversion of vegetable matter into good chyme is difficult or impracticable, and acetous fermentation is the consequence; but otherwise, for the gouty individual, I must approve of a mixed diet, of animal and vegetable food. Various facts serve to show that, in gout, the blood is overcharged with animal principles. All the secretions are loaded. It should be the patient's study not to exceed that quantity of food with which he can, by the exercise of self-control, just feel himself comfortable. Due regard is, at the same time, to be paid to the nervous energy; and this is a point always to be studied in the diet. It will often be expedient to make gradual rather than sudden changes."

Having thus concentrated all the chief points of this Letter, we shall merely observe that, although we differ from a writer and practitioner, of ample experience, on some minor points, yet we part with him entertaining a high opinion of his abilities in the treatment of that painful complaint which has occupied so much of his attention and observation.

---

ANATOMIE COMPARÉE DU SYSTEME NERVEUX CONSIDERÉ DANS SES RAPPORTS AVEC L'INTELLIGENCE, comprenant la description de l'encéphale et de la moelle rachidienne, des recherches sur le développement, le volume, le poids, la structure de ces organes chez l'homme et les animaux vertébrés; l'histoire du système ganglionaire des animaux articulés et des mollusques, et l'exposé de la relation qui existe entre la perfection progressive de ces centres nerveux et l'état des facultés instinctives, intellectuelles et morales. Par *Fr. Leuret*, Medecin de l'Hospice de Bicetre. Ouvrage accompagné d'un atlas de 33 planches in-folio, dessinées par M. Chazal et gravées sous sa direction. 1re Livraison—Tome Premier. Paris, chez J. B. Bailliere, 1839.

ATLAS—Première Livraison—Planches 1 à 8—Texte, Feuilles 1 à 4. J. B. Bailliere.

We look on this as a very valuable work. At present we shall content ourselves with merely pointing out the contents of the volume before us. At a future opportunity we shall present some account of our author's views.

He treats in succession of the nervous system of the molusca—of the articulata—of fish—of the cerebro-spinal nervous system of reptiles.

The plates which are uncoloured, but remarkably clearly executed, represent 1, the Nervous System of the Invertebrata—2, the Cerebro-Spinal Nervous System of Fish, Reptiles, and Birds—the Encephalon of the Mammifera whose Cerebral Lobes are deficient in Convolutions—4, the Encephalon of the Fox tribe—5, the Encephalon of the Cat tribe—6, the Encephalon of the Bear and Sable tribe—7, the Encephalon of the Sheep tribe, comprising the Ruminantia and Solipeda.

We recommend this work strongly to our anatomical readers.

